# Point-by-Point Pulsed Field Ablation Using a Multimodality Generator and a Contact Force–Sensing Ablation Catheter: Comparison With Radiofrequency Ablation in a Remapped Chronic Swine Heart

**DOI:** 10.1161/CIRCEP.123.012344

**Published:** 2022-11-23

**Authors:** Luigi Di Biase, Jacopo Marazzato, Fengwei Zou, Aung Lin, Vito Grupposo, Nilarun Chowdhuri, Jennifer Maffre, Salman Farshchi-Heydari, Tushar Sharma, Christopher Beeckler, Assaf Govari, Rahul Bhardwaj, Sanghamitra Mohanty, Andrea Natale, Hiroshi Nakagawa, Xiaodong Zhang

**Affiliations:** 1 Division of Cardiology, Albert Einstein College of Medicine at Montefiore Health System, NY (L.D.B., J.M., F.Z., A.L., N.C., X.Z.).; 2 Biosense Webster, Irvine, CA/Yokne’am, Israel (V.G., J.M., S.F.-H., T.S., C.B., A.G.).; 3 Loma Linda University Medical Center (R.B.).; 4 Texas Cardiac Arrhythmia Institute, St. David’s Medical Center, Austin (S.M., A.N.).; 5 Section of Cardiac Electrophysiology & Pacing, Robert and Suzanne Tomsich Department of Cardiovascular Medicine, Sydell and Arnold Miller Family Heart and Vascular Institute, Cleveland Clinic, OH (H.N.).

**Keywords:** atrial fibrillation, catheter ablation, electroporation, pulmonary vein, swine

## Abstract

**Background:**

Pulsed field ablation (PFA) has emerged as an alternative to radiofrequency ablation. However, data on focal point-by-point PFA are scarce. The aim of this study was to compare lesion durability and collateral damage between focally delivered unipolar/biphasic PFA versus radiofrequency in swine.

**Methods:**

Eighteen swine were randomized to low-dose PFA, high-dose PFA, and radiofrequency using a multimodality generator. Radiofrequency delivered by market-available generator served as control group. A contact force–sensing catheter was used to focally deliver PFA/radiofrequency at the pulmonary veins and other predefined sites in the atria. Animals were remapped postprocedurally and 28 days postablation to test lesion durability followed by gross necroscopy and histology.

**Results:**

All targeted sites were successfully ablated (contact force value, 13.9±4.1 g). Follow-up remapping showed persistent pulmonary vein isolation in all animals (100%) with lesion durability at nonpulmonary vein sites proven in most (98%). Regardless of the energy source used, the lesion size was similar across the study groups. Transmurality was achieved in 95% of targeted sites and 100% at pulmonary veins. On histology, PFA animals showed more mature scar formation than their radiofrequency counterpart without myocardial necrosis or inflammation. Finally, no sign of collateral damage was observed in any of the groups.

**Conclusions:**

In a randomized preclinical study, focally delivered unipolar/biphasic PFA guided by contact force values was associated with durable lesions on chronic remapping and with mature scar formation on histology without signs of collateral injury on necroscopy. Further studies are needed to investigate the long-term feasibility of this new approach to atrial fibrillation treatment.

**WHAT IS KNOWN?**

- Different from radiofrequency energy delivery, pulsed field ablation (PFA) uses nonthermal irreversible electroporation to induce cardiac cell death. The remarkable myocardial selectivity of this new energy source generally leads to well-defined myocardial lesions on histology.
- Although contact force proved to play a pivotal role in lesion formation during radiofrequency ablation, the role of catheter-tissue contact in creating adequate lesion formation is not clear during PFA.

**WHAT THE STUDY ADDS**

- In this randomized animal study, we compared lesion durability in animals randomized to radiofrequency versus PFA using different settings.
- This is the first implementation of a widely used contact force–sensing catheter in the setting of PFA.
- Despite the different histopathologic architecture, PFA and radiofrequency were associated with overlapping lesion durability and size on chronic evaluation provided that adequate contact force was maintained during the index ablation procedure.

Owing to its high selectivity for myocardial tissue,^1,2^ irreversible electroporation by catheter-based pulsed field ablation (PFA) has recently emerged as an alternative strategy to radiofrequency catheter ablation to effectively ablate atrial and pulmonary venous (PV) tissue.^1–5^

However, most PFA catheters have been designed in a “one-shot” manner^1–4^ which can limit the scope of ablation in case of extrapulmonary foci, variants of PV anatomy, or when precise linear lesion set is required to treat macro-reentrant atrial tachyarrhythmias or for substrate modification in persistent atrial fibrillation.^6^ Although an ablation strategy based on focally delivered PFA may provide sufficient versatility in these settings, data on the feasibility of point-by-point PFA delivered through a contact force (CF)–sensing catheter are scant.^7^

Therefore, in this randomized preclinical study, we compared durability of lesion formation on chronic 3-dimensional (3D) electroanatomic remapping using the CARTO system (Biosense Webster, CA) followed by histological evaluation in swine randomized to focally delivered PFA versus RF through the multimodality generator TRUPULSE 2 (Biosense Webster, CA) and the CF-sensing Thermocool SmartTouch ablation catheter (Biosense Webster, CA).

## Methods

The data that support the findings of this study are available from the corresponding author on reasonable request.

### Animal Description, Study Design, and End Points

Following quarantine, acclimation, and prescreen health assessment, 18 healthy swine were randomized to 4 study groups stratified by the type of energy source used (PFA versus radiofrequency) and the implemented generator (TRUPULSE 2 versus commercially available radiofrequency generator SMARTABLATE). By randomization scheme, PFA and radiofrequency applications were both delivered in a point-by-point fashion at specific anatomic sites in the right atrium (RA) and left atrium (LA) while maintaining adequate CF values (ie, 5–30 g). The study consisted of 4 arms described as follows:

- Group 1: Nominal dose PFA (ThermoCool SmartTouch ablation catheter with the multimodality TRUPULSE 2 generator).
- Group 2: High-dose PFA (ThermoCool SmartTouch catheter with the TRUPULSE 2 generator).
- Group 3: Test-radiofrequency delivered through the multimodality generator per current clinically approved radiofrequency settings (ThermoCool SmartTouch catheter with the TRUPULSE 2 generator).
- Group 4 or control: standard radiofrequency delivery (ThermoCool SmartTouch catheter with a market-available radiofrequency SMARTABLATE generator).

**Figure 1.**
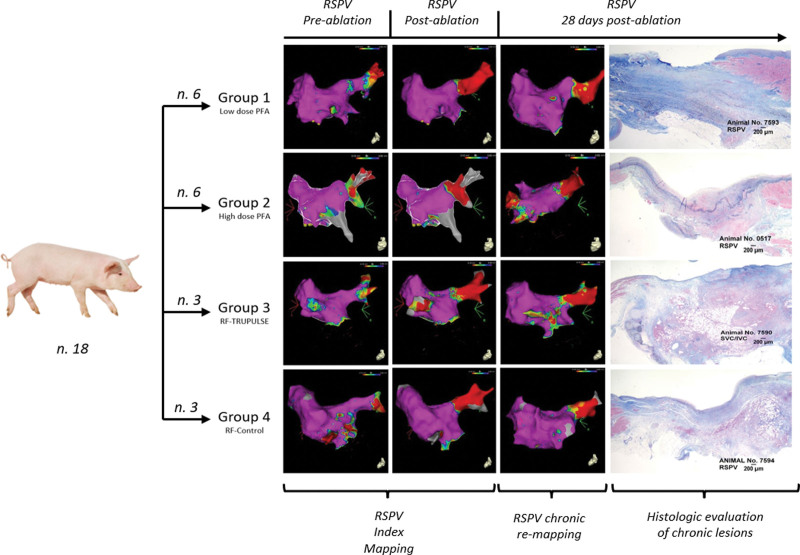
**Study design.** The animals were randomized to 4 study groups according to energy source (pulsed field ablation [PFA] vs radiofrequency [RF] energy) and generator used (multimodality generator TRUPULSE 2 vs market-available SMARTABLATE generator). The figure shows the example of right superior pulmonary vein (RSPV) ablation. After focally delivered PFA/RF, the pulmonary veins were mapped postprocedurally and 28+2 d after ablation to assess acute a chronic pulmonary vein isolation, respectively. The animals were then euthanized, and necroscopic assessment was performed to evaluate lesion size/histopathologic features and to search for any signs of collateral damage. PFA/RF ablation and the ensuing chronic evaluation to assess lesion durability were also performed at other predefined sites in the swine atria. See text for further details. LSPV indicates left superior pulmonary vein; and SVC-IVC, line connecting the superior to the inferior vena cava.

**Figure 2.**
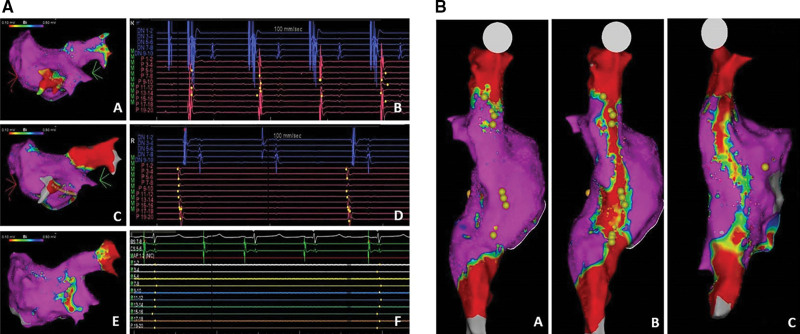
**Evaluation of lesion durability: acute and chronic pulmonary vein (PV) isolation and appraisal of lesion durability at nonpulmonary vein targets. A**, A pulsed field ablation (PFA) was delivered to the right superior PV (RSPV) in a swine randomized to nominal dose PFA (group 1). A 3-dimensional (3D) map of the left atrium (LA) was obtained preablation (**A**) with clear veno-atrial conduction through the RSPV on intracavitary signals recorded through a multipolar mapping catheter (**B**). RSPV tested isolated on remapping immediately after ablation (**C**) and dissociated PV potentials were recorded on the multipolar catheter placed inside the vein (ie, veno-atrial exit block; **D**). At 28 d after ablation, lesion durability was proven through chronic remapping confirming RSPV isolation (**E**) with no PV potentials inside the vein (**F**). Evaluation of lesion durability at atrial sites different from pulmonary veins (**B**). PFA was delivered to the posterior right atrium (RA), transecting the area between the superior (SVC) and inferior vena cava (IVC) in a swine randomized to nominal dose PFA (group 1). Before ablation, 3D mapping of the RA posterior wall was performed, and right phrenic nerve was identified by pacing (yellow dots; **A**). Point-by-point PFA was then delivered through a line connecting the SVC to the IVC that led to abatement of local bipolar potentials as identified by the red area of low bipolar potentials (<0.10 mV) on the voltage map (**B**). Thirty days after ablation, remapping of this area confirmed the abatement of the local bipolar potentials, thus proving lesion durability (**C**).

The study design is illustrated in Figure 1. The evaluated study end points are summarized in Table 1. In brief, the right superior PV and left superior PV, the LA posterior wall and LA roof, the mitral valve area or mitral isthmus (MVA), the cavotricuspid isthmus, and, finally, the area connecting the superior to the inferior vena cava were ablated in all animals. At these sites, acute efficacy and chronic lesion durability at 28±2 days postprocedure were evaluated on high-density CARTO mapping. Lesion durability was defined as permanent PVI in the evaluated PVs (Figure 2A) and as significant (or complete) abatement of local bipolar potentials on 3D voltage maps at the remaining atrial sites (Figure 2B). Then, lesion size and microscopic features were analyzed on gross necroscopy and histology and compared between the randomized arms. Finally, as outlined in Table 1, collateral damage to the neighboring myocardium and surrounding organs was investigated in the euthanized animals as well.

**Table 1.**
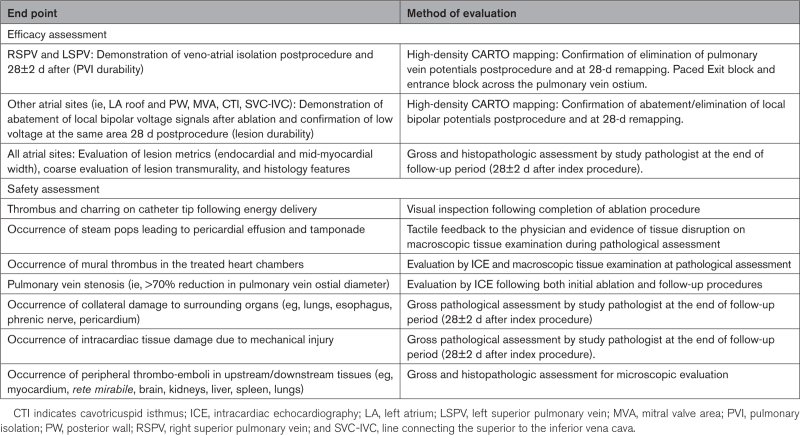
Study End Points

The porcine model is regularly used for preclinical testing for electrophysiology cardiac ablations due to the relative similarities to the human cardiac anatomy. The research protocol was approved by the Institutional Animal Care and Use Committee and conformed to the Position of the American Heart Association on Research Animal Use.

### Ablation Parameters

Table 2 summarizes the ablation parameters utilized in this study. PFA and radiofrequency were delivered through the multimodality generator TRUPULSE 2, which toggles between radiofrequency and PFA without catheter removal or cable switching. PFA lesions were delivered through trains of high voltage bipolar pulses of short duration, with each pulse being deployed as a square wave with a positive phase and negative phase separated by a brief delay. For the nominal dose recommended for clinical use, the TRUPULSE 2 generator was programmed to deliver 12 pulse applications (low-dose PFA for group 1) whereas 24 pulses (group 2) were used for high-dose PFA to test safety and gather dose-response information. The time between each pulse application was 1 s. However, radiofrequency ablations were delivered through TRUPULSE 2 (group 3 or Test-radiofrequency) or SMARTABLATE radiofrequency generator (group 4 or controls) with a target power of 30 to 50 W and irrigation flow rate of 8 to 15 mL/min (Table 2). In the 4 study groups, CF values ranged from 5 to 30 g during PFA/radiofrequency ablation at the predefined ablation sites in the swine atria.

**Table 2.**
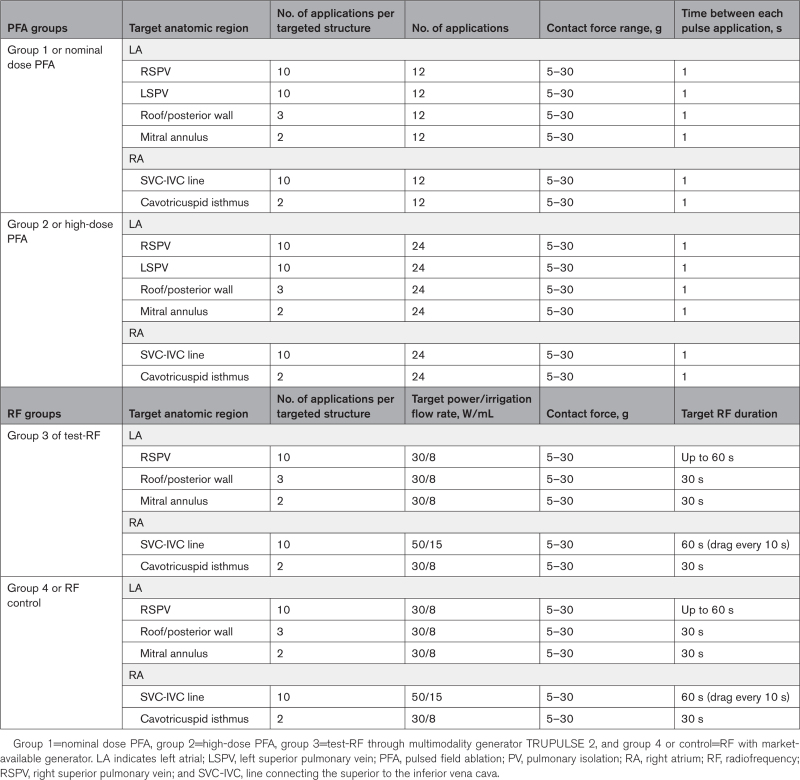
Ablation Parameters and Location

### Procedure Details

The investigated animals were premedicated, intubated, and mechanically ventilated after overnight fasting state. Femoral venous access was obtained percutaneously or via surgical cutdown. Intracardiac echocardiography (Acunav, Biosense Webster, CA) was utilized to rule out preexisting heart abnormalities, to guide the transseptal puncture, and to measure the ostial diameter and flow velocity of the PVs. The 3D rendering of the RA and LA was performed using dedicated electroanatomic mapping systems (CARTO, Biosense Webster) with the use of multipolar mapping catheters (PENTARAY, Biosense Webster, CA).

Before LA ablation, intravenous sodium heparin was administered to achieve an activated clotted time >350 s. Transseptal access was then obtained, and a 3D shell of LA was created with the PENTARAY mapping catheter. The right superior PV potentials were recorded in all animals followed by right superior PV isolation using PFA or radiofrequency according to the randomization scheme. The left superior PV was also assessed in 3 swine randomized to PFA groups, 1 to low-dose PFA (group 1) and 2 to high-dose PFA (group 2), respectively. High-density 3D remapping was then performed postablation, and PV ostial diameter was measured again with intracardiac echocardiography. Finally, PFA or radiofrequency spot ablations were delivered in the LA, including the roof, the LA posterior wall, and the MVA, to obtain significant abatement of the local bipolar voltage signals at these sites.

PFA and radiofrequency ablation were then performed at the RA posterior wall by superior to the inferior vena cava line, followed by 3 spot ablations on cavotricuspid isthmus. By study protocol, phrenic nerve pacing capture was required before and soon after PFA ablation to test its integrity.

### Follow-Up Procedure

Follow-up procedures were performed at chronic phase 28±2 days after index ablation. Using similar access techniques, lesion durability was tested at all study sites. Therefore, 3D remapping of LA and RA was created to obtain voltage maps in both chambers to confirm chronic PVI and abatement of local bipolar potential of the other LA and RA ablation sites. intracardiac echocardiography was used to reevaluate PV ostial flow and diameters. PV stenosis was defined as PV narrowing >70% of baseline measurement. Finally, the right phrenic nerve was rechecked by pacing to rule out chronic injury.

### Gross Anatomy and Histological Assessment

Upon termination of study, animals were euthanized, and gross necroscopy was performed. The heart, esophagus, brain, lungs, kidneys, liver, and spleen were grossly examined for evidence of injury and preserved in 10% neutral buffered formalin. Further samples were processed in a series of graded alcohols, embedded in paraffin blocks, sectioned via microtome at ≈5 micrometers, and mounted on glass slides. Standard hematoxylin and eosin as well as special staining with Gomori Elastin Trichrome for ablated areas in the heart were done.

At treatment sites, considering the thin-walled structure of the atrial chambers and the ensuing technical issues for the evaluation of the exact lesion depth achieved in the atria, only endocardial and myocardial lesion width were evaluated and compared between the investigated study groups. In addition, lesion transmurality was coarsely assessed at the targeted areas in all the study arms. A schematical representation of the analyzed lesion features is illustrated in Figure S1. Finally, the grade of myocardial necrosis, fibrosis, inflammation, and thrombus formation were assessed and compared between the study arms.

### Statistical Analysis

Continuous variables were expressed as mean±SD and the categorical variables as proportions. Differences in proportions between groups were tested using the χ^2^ test. Mean values of variables were compared by ANOVA, when appropriate. Analyses were performed using a significance level of α=0.05 (2-sided). Statistical analyses were performed using Medcalc.

## Results

### Periprocedural and Follow-Up Evaluation

PVs were isolated at first pass in all swine and confirmed at chronic remapping 28±2 day postablation (100%). A significant reduction of the amplitude of the local bipolar voltage signals was observed postprocedurally and on follow-up remapping at the remaining atrial sites aside from the MVA region where 2 swine randomized to radiofrequency-TRUPULSE (group 3) showed no postablation voltage abatement. Therefore, as a whole, 91 out of 93 targeted sites (98%) showed durable lesion formation on 28-day remapping.

Regardless of the energy source used, the average CF range maintained during ablation was 13.9±4.1 g with no statistical differences between different ablation sites (Table 3).

**Table 3.**
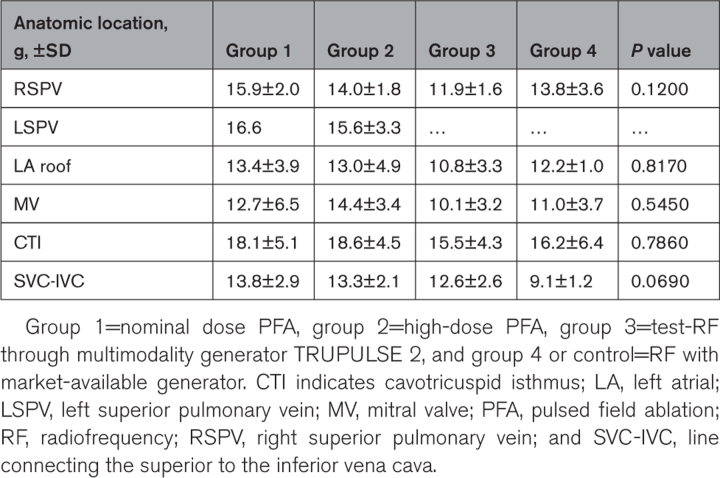
Contact Force Values at Target Sites According to Anatomic Location and Study Group

PV diameters and flow velocities had no significant change preprocedure and postprocedure in the investigated animals (*P*=0.713 and *P*=0.850, respectively). No pericardial effusion or any other major periprocedural complications were observed at the end of the procedure. No thrombus or charring was observed on catheter tip or in the treated heart chambers following delivery of PFA or radiofrequency energy. Per protocol, PFA was applied to the RA lateral and posterior wall and did elicit phrenic nerve response during ablation, confirming ablations on the nerve. Phrenic nerve remained intact after eliciting electrical stimuli as well as on gross necropsy and histopathologic examination.

### Gross Anatomy and Histological Assessment

Target (cardiac ablation sites) and nontarget tissues (brain, lungs, kidneys, rete mirabile, liver, phrenic nerve, spleen, tracheobronchial lymph node, esophagus, and untreated heart ventricles) were received from all swine for the macroscopic and microscopic evaluation.

No evidence of collateral damage in gross anatomy was observed. Specific to the protocol, no study-associated damage was noted in the phenic nerves, vagus nerves, tracheobronchial lymph nodes, aorta, or esophagus of any animal. In addition, no abnormalities were observed in any of the remaining tissues as per protocol, including absence of thromboembolism in the downstream and upstream tissue.

**Figure 3.**
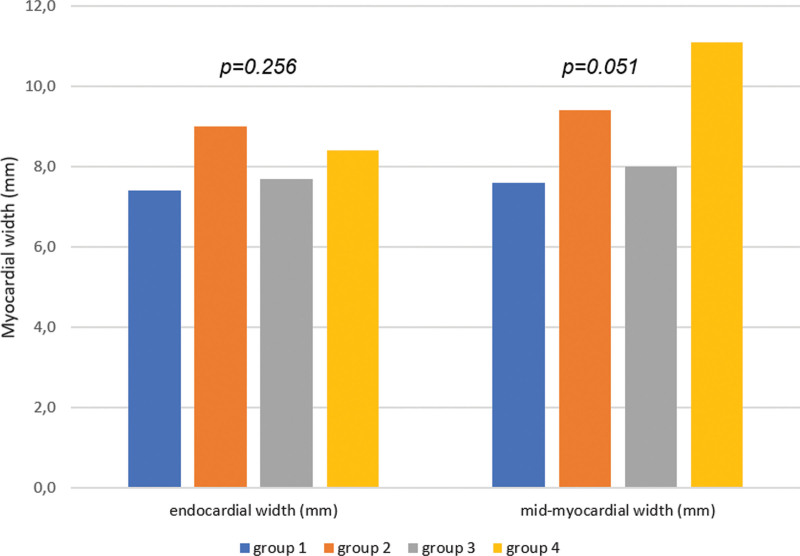
**Comparison of endocardial and myocardial lesion width between the investigated study groups.** Lesion width stratified by the investigated study groups. group 1=nominal pulsed field ablation (PFA); group 2=high-dose PFA; group 3=radiofrequency (RF) delivered by TRUPULSE generator; group 4=RF delivered by standard generator.

Independent of the energy source used, the observed mean endocardial and mid-myocardial width were 8.2±3.3 and 8.9±4.3 mm, respectively, with no difference between the randomized arms (Figure 3) even when stratified by the anatomic ablation site (Table 4). The lesion transmurality was achieved in 88 out of the 93 investigated atrial sites (95%), but 100% of PV lesions were transmural. Nontransmural lesions were observed at 5 atrial sites (4 at MVA and 1 at LA roof) in 4 animals (3 randomized to high-dose PFA and 1 to radiofrequency controls). In addition, 2 animals randomized to high-dose PFA (group 2) and Test-radiofrequency (group 3) and 1 swine randomized to radiofrequency controls (group 4) showed lesion mismatch between histology and gross necroscopy at the MVA and LA roof, respectively. In other words, at these sites, no lesions were detected on histology, whereas they were observed on gross necroscopy, thus suggesting incorrect specimen preparation in these specimens.

**Table 4.**
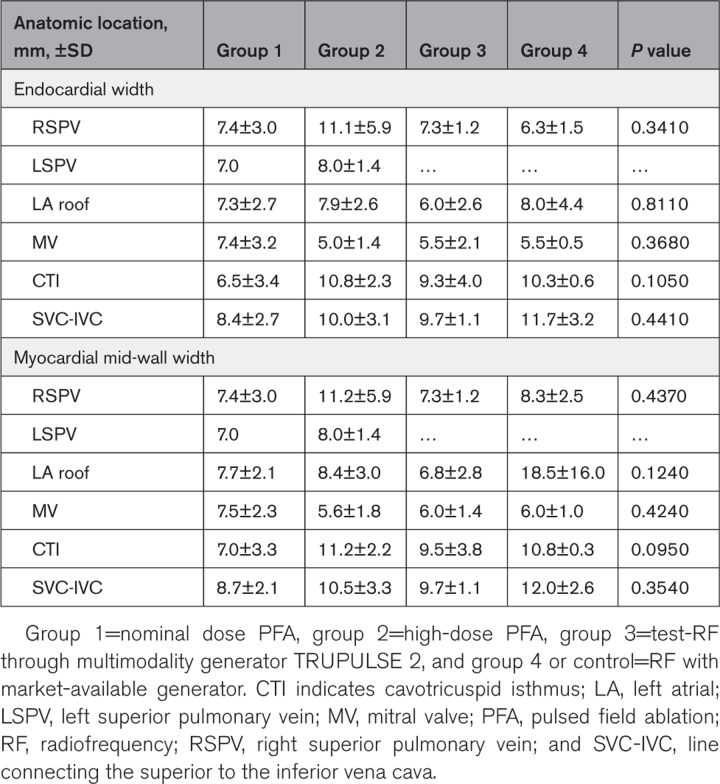
Lesion Width at Target Sites According to Anatomic Location and Study Group

**Figure 4.**
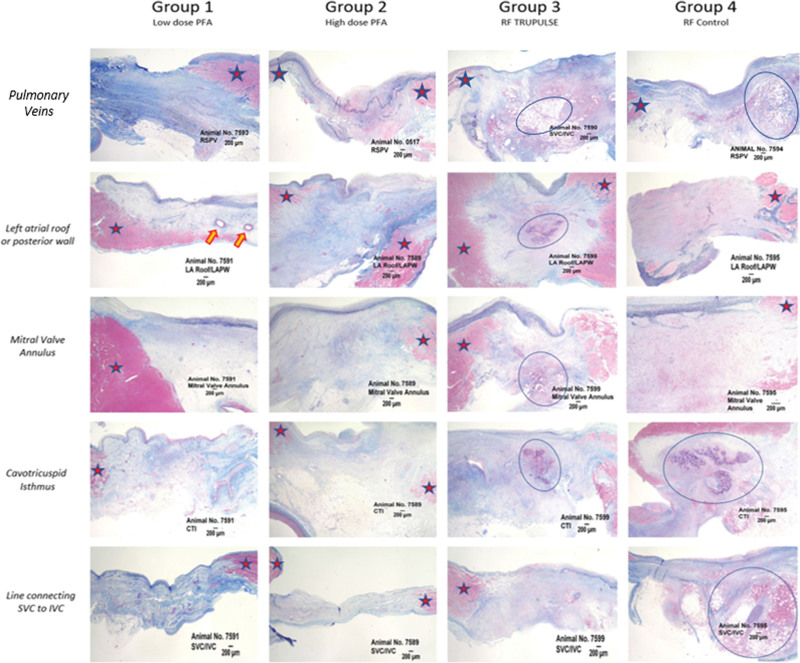
**Histological architecture of the evaluated lesions across different anatomic locations and different study arms.** Pulsed field ablation (PFA) vs radiofrequency (RF) lesions at the investigated ablated sites (pulmonary veins, left atrial roof or posterior wall, mitral valve annulus or mitral isthmus, cavotricuspid isthmus [CTI], and, finally, the posterior right atrium through a line connecting the superior vena cava [SVC] to the inferior vena cava [IVC]). Greater inflammatory response and necrotic burden were observed in animals treated with RF vs PFA. Red stars represent healthy tissue and blue circles areas of inflammation and necrosis. Orange arrows showing intact mid-myocardial vessels in PFA lesions. LA indicates left atrium; PW, posterior wall; and RSPV, right superior pulmonary vein.

Finally, the 4 study arms differed for the histopathologic architecture of the chronic lesions (Figure 4). PFA animals (group 1 and group 2) displayed a more mature scar with benign tissue reaction, whereas radiofrequency swine showed a greater burden of chronic inflammation and persistence of necrosis within the ablation region, especially when radiofrequency was delivered through the market-available radiofrequency generator (group 4 or controls). Of note, TRUPULSE 2–radiofrequency animals (group 3) showed intermediate features between PFA animals (group 1 and group 2) and radiofrequency controls (group 4). Mineralization was rare and only present in 1 animal in the low-dose PFA group (group 1).

## Discussion

Radiofrequency is considered the cornerstone for atrial fibrillation ablation. However, lesion formation is influenced by multiple factors in this setting.^6^ Furthermore, the potential damage to surrounding organs is a well-known crucial issue limiting radiofrequency ablation,^6^ often precluding adequate catheter-tissue contact. Although PFA has recently emerged as a promising treatment option due to irreversible electroporation myocardial selectivity,^1,2^ data on point-by-point PFA energy delivery are scarce in this setting.^5^

To our knowledge, this randomized, preclinical study represents the first evidence of the feasibility of focally delivered PFA versus radiofrequency using the CF-sensing Thermocool SmartTouch ablation catheter implemented with the multimodality generator TRUPULSE 2. This represents a promising strategy since the system toggles between radiofrequency and PFA without catheter removal or cable switch and allows for the utilization of 3D electroanatomic mapping systems, thus limiting fluoroscopic exposure in either setting.

In this study, biphasic/unipolar PFA delivered through the TRUPULSE 2 system and guided by CF values showed similar periprocedural feasibility and durable lesion formation 28 days postablation on 3D CARTO voltage maps as compared with radiofrequency ablation with the market-available SMARTABLATE generator. No collateral damage was observed on gross anatomy even when high-dose PFA settings were utilized. As for the myocardial targeted sites, the average lesion size was comparable on histology between the explored anatomic regions in the investigated study groups. In addition, the TRUPULSE 2 generator helped to achieve complete lesion transmurality at most targeted sites regardless of the energy source used.

Finally, consistent with other studies conducted with “one-shot” technologies,^3,5,8^ we observed that focally delivered PFA lesions were associated with more mature lesion formation and generally displayed scarcer inflammatory response and necrosis than radiofrequency lesions on chronic histology evaluation.

The optimal ablation settings are yet to be established during PFA.^9^ Testing a 9 mm lattice-tip catheter that focally delivered PFA and radiofrequency on swine thoracic veins, Koruth et al^5^ recently showed that durable lesion formation significantly improved for high-dose PFA compared with low-dose PFA without PV stenosis nor inflammation or necrosis. However, in this study, the overall lesion size was comparable at the explored treatment sites regardless of the PFA ablation protocol used. However, despite similar lesion size, 50% of swine randomized to high-dose PFA (group 2) displayed nontransmural lesions at the mitral isthmus site. Although the remarkable thickness of this complex anatomic structure would partly explain these findings, the reason why it was more commonly observed in high-dose PFA is merely speculative. Moreover, 2 swine randomized to radiofrequency-TRUPULSE 2 group showed missing lesions on histology or absence of significant abatement of local bipolar voltages at the mitral isthmus region on 28-day remapping as well. Therefore, further studies are needed to investigate the best PFA/radiofrequency ablation settings through the TRUPULSE 2 system to effectively ablate this complex anatomic structure.

Finally, the role of CF during PFA is still under debate. Although we observed that adequate maintenance of CF values at different ablation sites helped achieve similar lesion durability regardless of the energy source used, Mattison et al^7^ found that biphasic focal PFA has minimal effects on acute lesion dimensions despite a wide range of applied CF values (0 to 50 g) in a porcine perfused model, thus suggesting that tissue contact is more important than the magnitude of CF when PFA is focally delivered. However, other recent evidence would prove otherwise.^10^

In light of this evidence, unipolar/biphasic PFA focally delivered through the TRUPULSE 2 generator and the CF-sensing Thermocool SmartTouch ablation catheter seem a feasible alternative to standard point-by-point radiofrequency delivery for ablation of different LA and RA substrates in swine, provided that adequate CF values are kept during ablation.

Although the randomization scheme represents one of the major strengths of this study, some study limitations should be acknowledged. First, this study has a small sample size and thereby it might be potentially underpowered to detect any statistical difference between the randomized study arms. Second, as the study was conducted on the atria, the accurate estimate of lesion depth could not be performed in these thin-walled atrial structures and thereby only lesion width was measured and transmurality coarsely assessed in this study. Therefore, future studies are required to better explore the impact of different PFA settings on lesion depth and tissue transmurality in thicker anatomic structures, such as the ventricular chambers. Then, we only explored specific PFA/radiofrequency settings delivered through a dedicated multimodal PFA/radiofrequency generator and CF-sensing ablation catheter. Hence, further studies are required to investigate different PFA settings using different ablation catheters and generators. However, focally delivered high-dose PFA (ie, 24 pulses) displayed mature chronic healing and similar lesion size compared with standard radiofrequency with no evidence of collateral damage, including phrenic nerve palsy.^11^ Whether high-dose PFA should be the preferred treatment option in this setting is yet to be established and broader, prospective studies are required to address this point.

## Conclusions

When adequate catheter-tissue contact is established at different atrial sites in swine, focally delivered unipolar/biphasic PFA proved a feasible and alternative ablation strategy to point-by-point radiofrequency, showing durable lesion formation on chronic remapping and mature scar formation on histology with no signs of collateral damage. However, broader and prospective studies are required to investigate the optimal ablation parameters of focally delivered PFA and the role of CF to guide PFA in this setting.

## ARTICLE INFORMATION

### Sources of Funding

The study was supported by Biosense Webster.

### Disclosures

Dr Di Biase is a consultant for Stereotaxis, Biosense Webster, Boston Scientific, Abbott Medical, and I-Rhythm and has received speaker honoraria/travel from Medtronic, Atricure, Biotronik, Baylis Medical, and Zoll. V. Grupposo, Drs Marazzato, Farshchi-Heydari, Sharma, C. Beeckler, and Dr Govari are Biosense Webster Employees. The other authors report no conflicts.

### Supplemental Material

Figure S1

## Supplementary Material
