# Supplementary material for: Point-by-Point Pulsed Field Ablation Using a Multimodality Generator and a Contact Force–Sensing Ablation Catheter: Comparison With Radiofrequency Ablation in a Remapped Chronic Swine Heart
Source: Circ Arrhythm Electrophysiol. 2022 Nov 23;16(12):663–71. doi: 10.1161/CIRCEP.123.012344 (PMC10734778; doi:10.1161/CIRCEP.123.012344)
Supplement: Supplementary file 1 [file hae-16-663-s001.pdf]

SUPPLEMENTAL MATERIAL

Supplemental Figure I

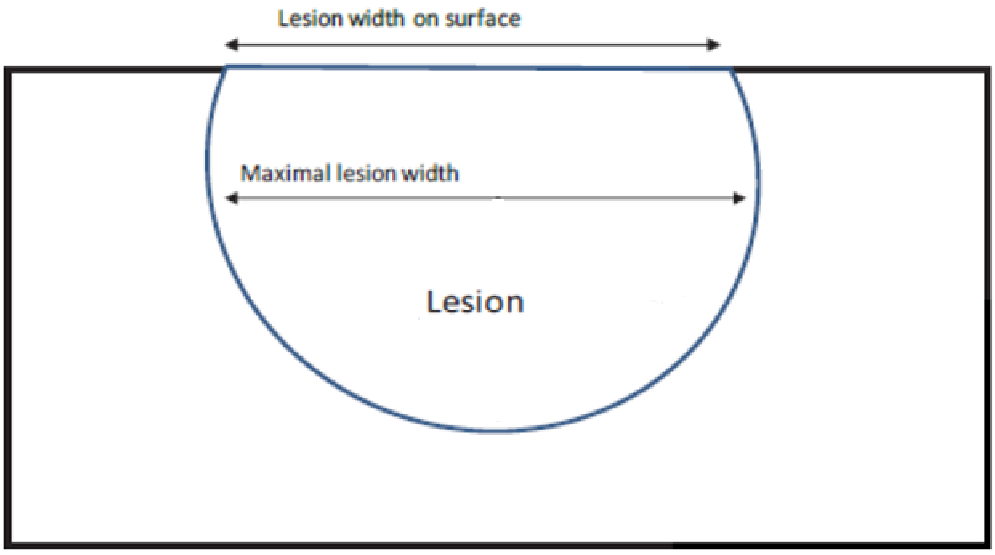

Supplemental Figure I Legend.

Schematical representation of the evaluated lesion metrics. After 28 ± 2 days post ablation, lesion metrics – endocardial and mid-myocardial lesion width – were evaluated at each investigated site on gross necroscopy and histology.
