# Supplementary figures and images for: Point-by-Point Pulsed Field Ablation Using a Multimodality Generator and a Contact Force–Sensing Ablation Catheter: Comparison With Radiofrequency Ablation in a Remapped Chronic Swine Heart
Source: Circ Arrhythm Electrophysiol. 2022 Nov 23;16(12):663–71. doi: 10.1161/CIRCEP.123.012344 (PMC10734778; doi:10.1161/CIRCEP.123.012344)

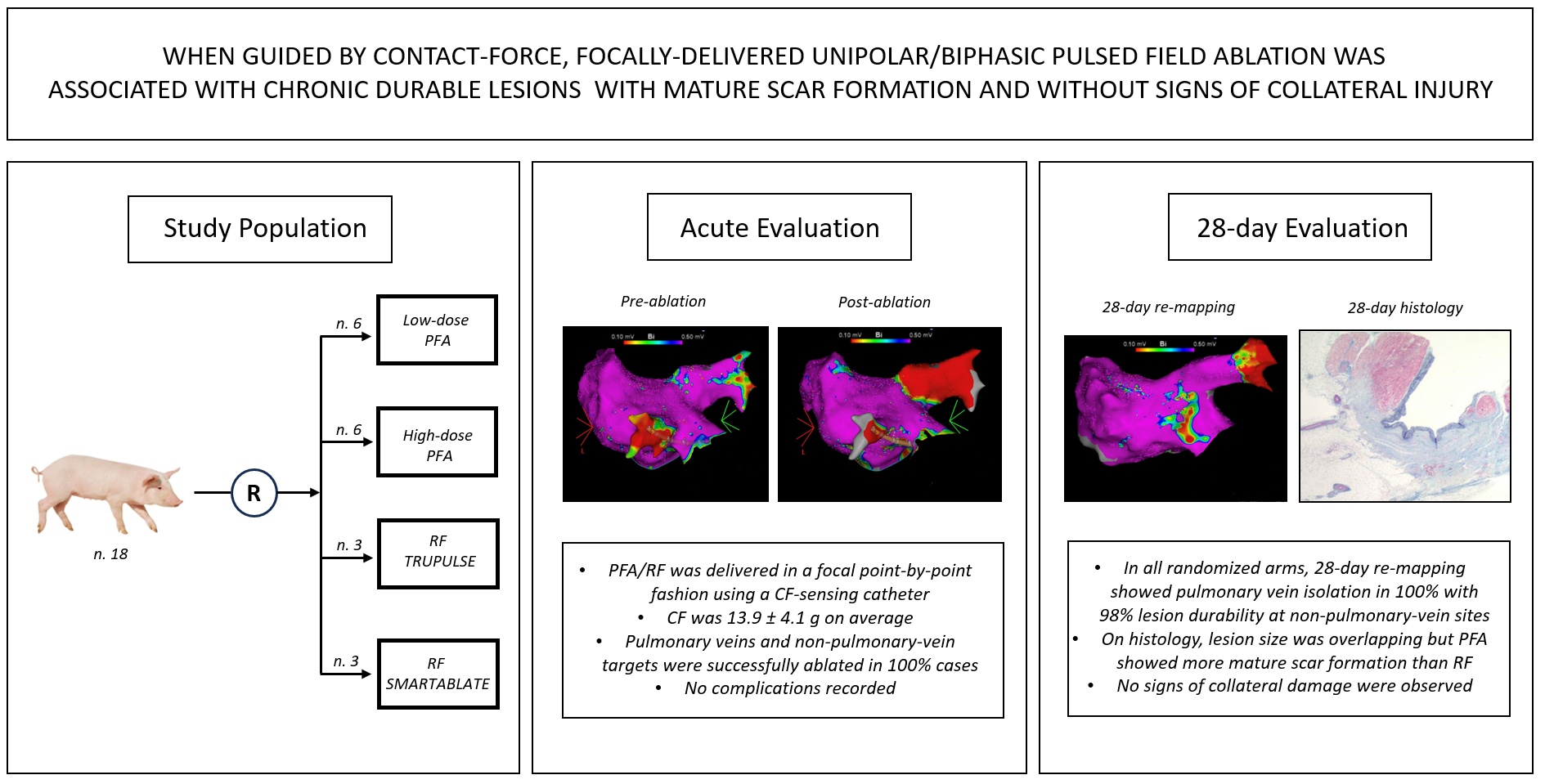

Supplement: Supplementary file 2 [file hae-16-663-s002.tif]
